# Classification of human walking context using a single-point accelerometer

**DOI:** 10.1038/s41598-024-53143-8

**Published:** 2024-02-06

**Authors:** Loubna Baroudi, Kira Barton, Stephen M. Cain, K. Alex Shorter

**Affiliations:** 1 Mechanical Engineering, University of Michigan, Ann Arbor, 48109 USA; 2 Robotics, University of Michigan, Ann Arbor, MI 48109 USA; 3 Chemical and Biomedical Engineering, West Virginia University, Morgantown, WV 26505 USA

**Keywords:** Engineering, Computer science

## Abstract

Real-world walking data offers rich insights into a person’s mobility. Yet, daily life variations can alter these patterns, making the data challenging to interpret. As such, it is essential to integrate context for the extraction of meaningful information from real-world movement data. In this work, we leveraged the relationship between the characteristics of a walking bout and context to build a classification algorithm to distinguish between indoor and outdoor walks. We used data from 20 participants wearing an accelerometer on the thigh over a week. Their walking bouts were isolated and labeled using GPS and self-reporting data. We trained and validated two machine learning models, random forest and ensemble Support Vector Machine, using a leave-one-participant-out validation scheme on 15 subjects. The 5 remaining subjects were used as a testing set to choose a final model. The chosen model achieved an accuracy of 0.941, an F1-score of 0.963, and an AUROC of 0.931. This validated model was then used to label the walks from a different dataset with 15 participants wearing the same accelerometer. Finally, we characterized the differences between indoor and outdoor walks using the ensemble of the data. We found that participants walked significantly faster, longer, and more continuously when walking outdoors compared to indoors. These results demonstrate how movement data alone can be used to obtain accurate information on important contextual factors. These factors can then be leveraged to enhance our understanding and interpretation of real-world movement data, providing deeper insights into a person’s health.

## Introduction

Walking is a fundamental human movement that has many benefits for mental and physical health^1–4^. With the advancements of micro-electromechanical systems (MEMS), researchers are now able to measure human motion outside the lab for extended periods. Real-world measurements are often conducted over long periods where there is little to no control over or explicit knowledge of a participant’s behavior. Generally, people engage in numerous activities across different contexts in their daily life. Various methods have been developed to gather information about what an individual is doing. Human activity recognition (HAR) serves as the initial step in understanding real-world data as it allows for the classification of an individual’s activities and the identification of walking instances. HAR research has successfully utilized different combinations of wearable sensors (such as mobile phones and inertial measurement units) and methods (including classic machine learning models and deep learning) to achieve accurate classification performance^5–9^. Research has also been conducted in the field of transportation mode detection using mobile phones and wearable sensors^10,11^. However, even within the walking activity itself, there exists a range of contexts that give rise to different behaviors.

There are many factors that can cause changes in gait. Firstly, the location where an individual is walking has a significant impact on their kinematics. Different terrains have been shown to influence how people negotiate their walk^12,13^. The location where someone walks can provide insight into their habits, such as whether they explore beyond their home and engage with the community^14^. Additionally, certain features of the built environment that individuals navigate can impact their mobility^15,16^. These various factors are directly related to health and well-being and therefore important to monitor. Secondly, the purpose of a walk can result in different walking strategies, even when individuals are walking in the same location^17^. For example, people tend to walk faster when commuting compared to a leisurely walk, despite both taking place outdoors in similar locations. Thirdly, there are internal factors that can affect human movement, such as mood. Contrasting moods, like happiness versus sadness, can lead individuals to exhibit different walking behavior^18–20^. Overall, acquiring information about these factors is crucial for understanding any observed variability in real-world walking.

The utilization of GPS data has proven to be successful in answering various research questions related to real-world human movement and mobility^21–23^. Kim et al. found that both lower-limb amputees and non-amputees tend to walk faster when they are away from their homes^14^. Similarly, Baroudi et al. recently quantified differences in walking speed for individuals walking in different real-world contexts, such as work, home, or commuting^17^. These studies used either dedicated GPS receivers or leveraged the GPS capabilities of mobile phones. However, GPS receivers can be cumbersome to use over extended periods as they require frequent recharging, cause privacy concerns, and add an extra device for individuals to carry; mobile phones do not offer the same level of resolution and can result in sparse data that is challenging to utilize effectively. Another tool researchers have employed for gaining insights into an individual’s real-world context is self-reporting^24,25^. Self-reporting enables the collection of more detailed data, but it heavily relies on the participant’s compliance and often leads to incomplete datasets. Furthermore, self-reporting is burdensome and not practical for extended periods of data collection. Cameras offer arguably the most direct means of gathering information about a person’s whereabouts. Doherty et al. used both a camera and an accelerometer to objectively quantify real-world activity^26^. Researchers have also developed accurate frameworks for the classification of camera data to obtain information on terrain types and surface inclines^27^. However, the use of camera data can raise privacy concerns, especially when used over extended periods. Practicality is another consideration as individuals need to carry the camera, keep it charged, and ensure there are no obstructions. Overall, although these methods are advantageous in many aspects, factors such as practicality, participant burden, and privacy need to be taken into account for real-world data collection.

Accelerometer-based methods have emerged as an alternative for analyzing real-world data with regard to context. Hu et al. used a single inertial measurement unit (IMU) on the lower back to differentiate between flat and uneven terrain, as well as distinguishing between older and younger participants^28^. Hashmi et al. used IMUs embedded in a smartphone, placed on the lower back and chest, to classify various terrain features^13^. While both studies demonstrated the feasibility of using IMUs to accurately classify terrain, the datasets used were created in a controlled environment, with participants walking at steady state on selected surfaces. This synthetic aspect of data collection may limit the ecological validity of the classifiers in real-world scenarios. Additionally, the sensor placements used in these studies may restrict the practical implementation of these solutions over extended periods.

Hashmi et al. also included a classification of indoor vs. outdoor environments^13^, which can provide important insights for clinical decision-making. Understanding the proportion of time an individual spends indoors can be indicative of lifestyle choices and mental health. Outdoor walking, often more challenging, can be particularly useful for the assessment of certain patient groups’ mobility. Conversely, indoor walking occurs in a more controlled environment that can be replicated in the lab. As such, differentiating movement in these two environments can provide insight into an individual’s health and well-being. Ali et al. proposed SenseIO, an accurate framework that combines different mobile phones modalities (e.g., Wi-Fi, accelerometer, proximity, light, and time-clock) for environmental classification (indoor vs. outdoor)^29^. However, this method suffers from a high consumption of smartphone energy. Kelishomi et al. propose an alternative approach that leverages smartphone motion sensors to help detect whether an individual is moving indoors or outdoors^30^. While the classification results were accurate, the dataset used to train and evaluate the algorithm was synthetic and may not be a good representation of real-world scenarios.

In this study, we propose an approach to identify walking context in the real world utilizing a single thigh-worn accelerometer. Our study makes the following contributions:

- *Algorithm development* We developed a classification algorithm that leveraged the natural grouping of real-world walking into bouts to identify walks indoors versus outdoors.
- *Validation of our algorithm with a real-world dataset* To train and validate the model, we used a dataset generated from a data collection on 20 participants in the real world over a week, where GPS and self-reporting information were collected to label the different walks.
- *Analysis of differences in walking kinematics with an extended dataset* Once validated, we used our model to label indoor and outdoor walks from a different dataset, where 15 participants were equipped with the accelerometer over two consecutive weeks. Finally, we characterized the influence of walking indoors versus outdoors on walking kinematics.

This novel approach has the potential to facilitate the parsing and analysis of real-world walking data while utilizing only an accelerometer.

## Methods

### Overview

**Figure 1:**
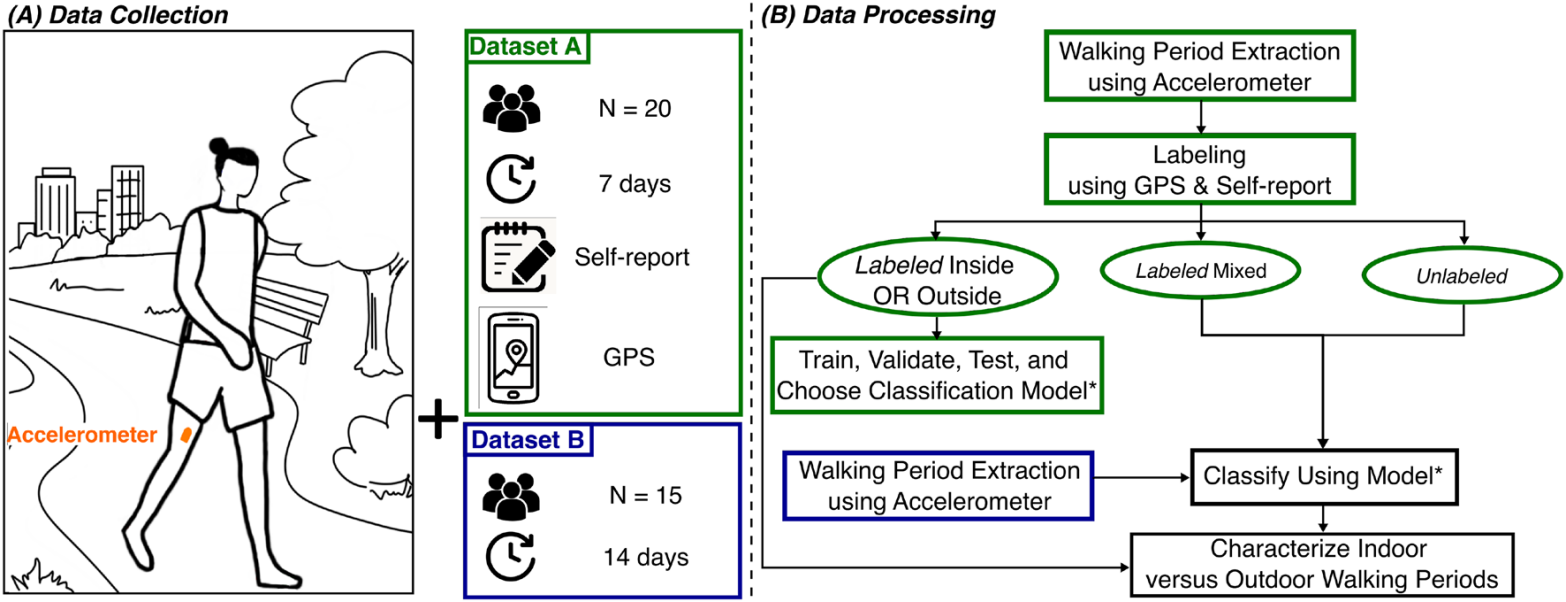
Data collection and processing overview—**(A)** Dataset A was collected over 7 days on 20 participants, using an accelerometer, self-report, and GPS data from the participants’ phones. Dataset B was collected over 14 days on 15 participants using an accelerometer. **(B)** The walking periods from dataset A were labeled using the GPS and self-report data. Walking periods labeled exclusively indoor or outdoor were used to train, validate, and test a classification model. The walking periods labeled mixed (e.g., inside and outside), as well as all the unlabeled walking periods from dataset A and B were classified using the model to characterize indoor versus outdoor walks.

Figure 1 shows an overview of the data collection and processing framework. We leveraged two datasets in this study: datasets A and B. All methods were carried out in accordance with relevant guidelines and regulations. For both datasets, the University of Michigan’s Institutional Review Board approved the procedures. Every participant gave their informed consent, before the studies commenced. Dataset A was collected using a thigh-worn accelerometer, self-report, and GPS data over 7 days with 20 participants. Participants were asked to report the purpose and location of the walks carried out throughout their day. Dataset B was collected using only the thigh-worn accelerometer over 14 days with 15 participants. We used the self-report and GPS data to label the walking periods from Dataset A. The walking periods labeled exclusively inside or outside were used to train, validate, and test a classification model. Then, we used the classification model to label the walking periods labeled as mixed (e.g., inside and outside), as well as the unlabeled walking periods from Dataset A, and all the walking periods from Dataset B. Finally, all the labeled walking periods from both Dataset A and B were used to characterize the differences between indoor and outdoor walking periods.

### Datasets

The details for the data collection of Dataset A and B can be found here^17,31^. Briefly, for Dataset A, we collected data on 20 participants over a week in the real world (Table 1). 13 females and 7 males between 21 and 49 years old with an average of 26.1 were recruited from a population of students during the summer in Ann Arbor, Michigan. The participant aged 49 years was a non-employed adult. Contextual information was collected using self-reporting and GPS data from their phones using an app called Ethica app (Ethica Data [Toronto, Canada]). For the self-reporting, participants were asked to maintain an activity log describing where they walked (ex: home, work, etc.) and the purpose of their walk (ex: going to work). We also conducted an exit interview after the real-world data collection to ensure that the self-reporting was complete and accurate. Motion data was collected using the activPAL (activPAL^TM^ [PAL Technologies Ltd., Glasgow, UK]), a thigh-worn accelerometer. This device can be placed on the thigh using tape and offers 2-week continuous monitoring. It samples at 20Hz and possesses a 3-axis accelerometer ($range = \pm 4 \,{\text {g}}$). The sensor’s size and battery life allow for an unobtrusive placement and high compliance. Additionally, the proprietary algorithm of the sensor offers an accurate classification of activities that we used to isolate walking^32,33^. Dataset B was collected on 15 participants over 2 weeks in the real world, using only the activPAL (Table 1). All the participants were students between 20 and 30 years old. It is important to note that 4 subjects are in both datasets. Additionally, although dataset B was over a longer period, the data collection period was amidst the COVID-19 pandemic, which might lead to a decrease in measured activity. Information about fitness and occupation were not part of the exclusion criteria and were not collected.

**Table 1:** Datasets details.

	Dataset A	Dataset B
Sample size	20	15
Age	$\mu =26.1$ $\sigma = 6.1$	$\mu = 25.3$ $\sigma = 2.1$
Male:female	6:11	7:8
Measurements	activPAL Self-report GPS	activPAL
Duration	7 days	14 days

### Classification algorithm development

**Figure 2:**
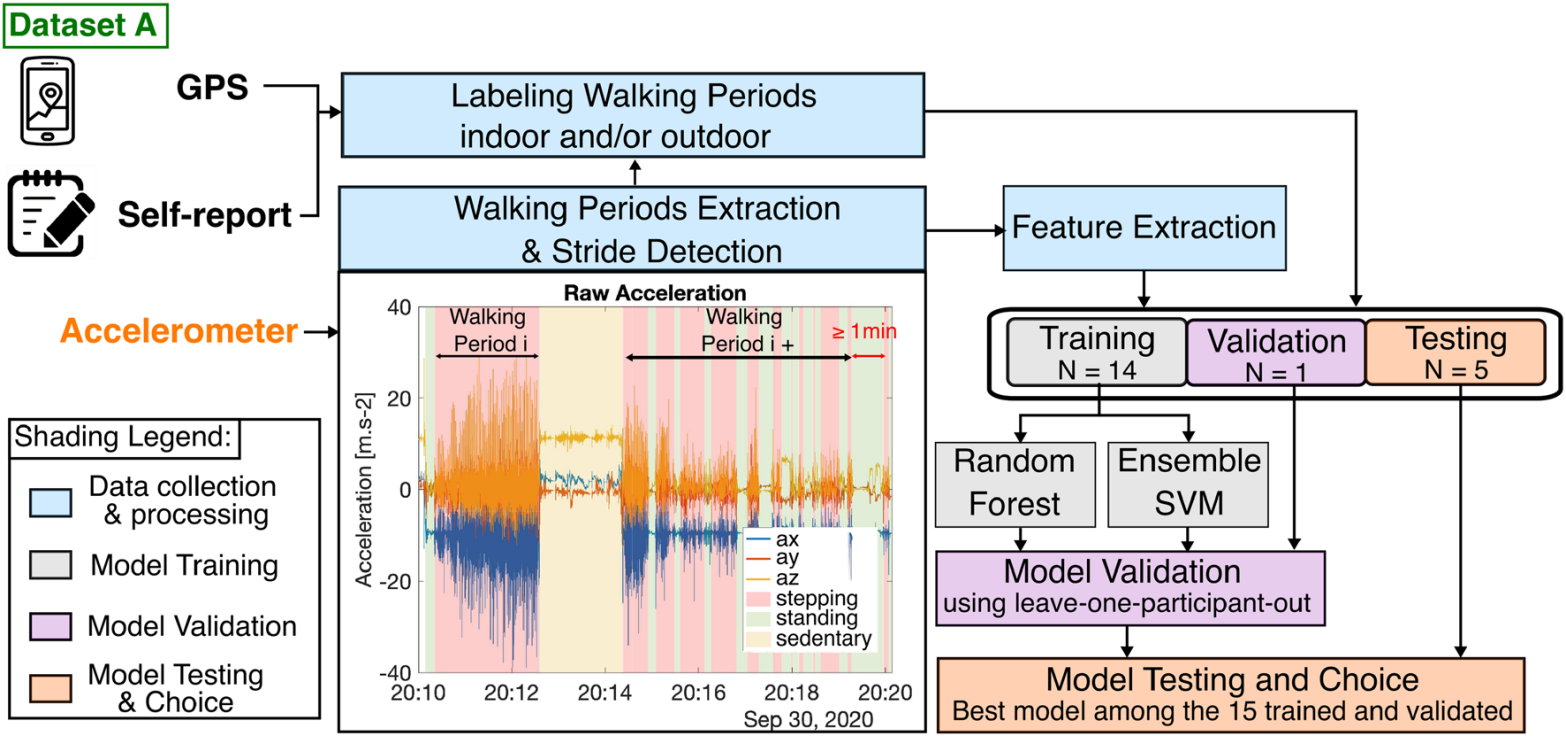
Classification model—We extracted walking periods from the accelerometer that we then labeled using self-report and GPS data. We extracted features from the accelerometer data and the stride detection. Labeled walking periods were then split into training, validation, and testing set. We trained and validated two different learning algorithms, Random Forest and Ensemble SVM, using a leave-one-participant-out scheme. This led to 15 trained models that were then tested on the 5 remaining untouched participants’ data. The best-performing model was chosen for the rest of the analyses.

Figure 2 shows the data processing framework we used to create the algorithm to classify walking periods into outdoor or indoor.

#### Walking period extraction

Walking in the real world can be understood as an ensemble of walking bouts. However, there are bouts of walking that are likely to belong to a same walking activity. For instance, if an individual stops at a pedestrian light, the walks before and after that stop could be grouped together. We introduced the notion of walking period to capture this start-and-stop dynamic of real-world walking. This method was created and outlined in earlier work^31^. Briefly, we used the activPAL to identify stepping bouts. Then, bouts that were separated by a standing period of less than 1 min were grouped together into a stepping period. Lastly, we developed a classification algorithm to extract walking periods from these stepping periods, since the activPAL does not distinguish between walking and running^31^.

#### Data labeling

**Figure 3:**
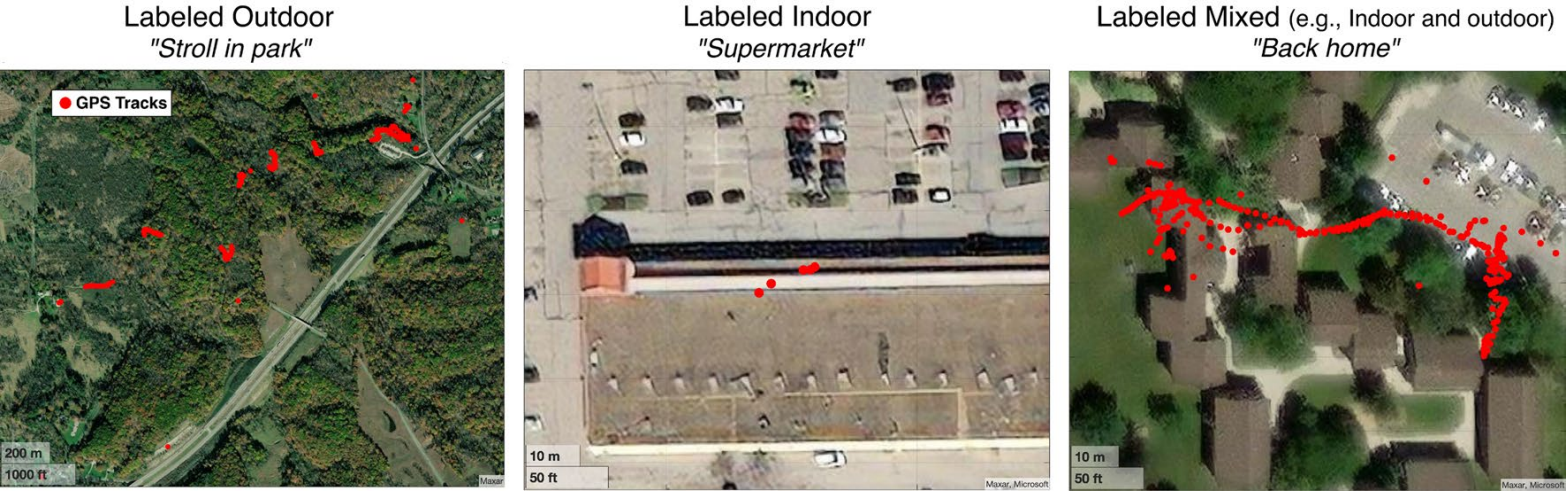
Data labeling using GPS and self-report data—We show here 3 examples for walking periods labeled indoor, outdoor, and both indoor and outdoor. The GPS sampling was variable and dependent to the type of phone used. The text in quotations corresponds to the participant’s self-report.

We manually labeled the walking periods from Dataset A using the self-report and GPS data. We combined a visualization of the GPS data with a satellite map and the information given in the participants’ self-report to assign either an inside or outside label, or a mixed label when it appeared participants were walking both inside and outside (Fig. 3). There are walking periods that were not labeled, either because there was no associated GPS data, because the self-report was missing, or both.

#### Feature extraction

We used a custom algorithm to extract strides from the thigh-worn accelerometer. Using the timing between strides, we computed stride time and stride frequency for each stride in a walking period. Then, we first extracted what we named the biomechanics feature set, based on our domain knowledge. This set includes walking period duration, walking period continuity (e.g., proportion of total standing time within a period, with 100 being no standing time), mean and standard deviation of stride frequency. Stride frequency was normalized by $\sqrt{g \cdot l_0}$, with $l_0$ being leg length^34^. Leg length was measured from the anterior superior iliac spine to the floor. Walking period continuity was defined as:

1 $$\begin{aligned} period \; continuity = \frac{period \; duration - standing \; time}{period \; duration} \times 100. \end{aligned}$$

This reduced feature set was selected because of the demonstrated relationship between walking period duration and continuity and walking variability^31^. Stride frequency is also a key parameter that is likely to vary with the environment. Moreover, this feature set can be derived using other accelerometer-based sensors with different body placements. Additionally, we computed 20 other features from both stride frequency and the raw accelerometer signal. These other features were selected based on the existing literature^31,35,36^. We compared model performance using both feature sets to identify features best capable of distinguishing between different walking environments. A table describing the features is available in the Supplementary Material.

#### Classification model

##### Learning algorithms and hyperparameters

We compared the performances of two supervised ensemble learning algorithms that use different classification principles to separate indoor versus outdoor walking periods: Ensemble Support Vector Machine (SVM)^37^ and Random Forest^38^. We used ensemble methods to solve the issue of imbalanced classes but also improve the generalizability of our model^39^. Ensemble methods combine multiple base models to improve classification performances. Both SVM and Random Forest algorithms have successfully been used for terrain classification tasks with both humans and robots^13^. We used grid search to optimize the hyperparameters of both algorithms. The ensemble SVM we used is a bagging (e.g., bootstrap aggregation) classifier with SVM as a base model, 60 estimators, and a radial basis function kernel. The Random Forest algorithm used 40 estimators and used bootstrapping to build the trees. We trained both algorithms on the set of biomechanics features and all features. In summary, we evaluated 4 different cases: Ensemble SVM with biomechanics features, Ensemble SVM with all features, Random Forest with biomechanics features, and Random Forest with all features. We used the Scikit-learn library (version 1.0.2) from Python to train and evaluate the different learning algorithms^40^.

##### Model training, validation, and testing

To build our model, we only used walking periods labeled exclusively indoor or outdoor (Fig. 1). We divided our dataset into training, validation, and testing sets. The training and validation set contained walking periods from 15 participants and followed a leave-one-participant-out method to tune the hyperparameters of our models and evaluate the generalizability to new participants. This means that we trained a model on data from 14 participants and validated it on the remaining participant. This process was iteratively performed 15 times, with each participant serving as the validation set once, and for each of the four cases described in the previous paragraph (2 algorithms $\times$ 2 features sets). This provided 15 models that we evaluated and we chose the best-performing case to use on the 5-participant testing set. For instance, if the average performance of the models (e.g., average accuracy, f1-score, and AUROC) that were trained and validated using Ensemble SVM on all features is the highest, we will use the 15 models from this case on the testing set. The model that performed the best (e.g., highest accuracy, f1-score, and AUROC) on the testing set among the 15 trained models for the best case was chosen for the classification of the walking periods labeled as mixed and the unlabeled walking periods of dataset A, as well as all the walking periods in dataset B (Fig. 1).

##### Model evaluation

We used different metrics to evaluate our model and represent its performances both during training and testing in the different cases and for the different models. First, we used accuracy to determine the overall proportion of correctly classified walking periods. We complemented accuracy with F1-score since accuracy can be misleading when dealing with imbalanced datasets. F1-score takes into account both false positives and false negatives to ensure that the performance is not biased towards the more frequent class. Finally, we also used Area Under the ROC Curve (AUROC) to measure the model’s ability to separate between indoor and outdoor classes. AUROC is also useful for imbalanced datasets as it takes into account false positives and false negatives. The combination of these different performance metrics provides a more comprehensive picture of the model’s performance.

##### Feature importance

We used feature permutation to estimate the importance of each feature in the performances of our model. This method consists of randomly shuffling the values of a given feature in the validation set and calculating the change in performance of the model with the shuffled data. We used the test set to evaluate feature importance.

##### Analysis of mixed walking periods, labeled indoor and outdoor

We did not use walking periods that happened both inside and outside to train our model to avoid decreasing the model’s ability to distinguish between indoor and outdoor. However, we investigated how the chosen model classified these mixed walking periods. The researcher used both the GPS data and the participant’s self-report to label as best as possible the mixed walking periods based on whether they appeared to be mostly indoor or mostly outdoor. Then, we investigated whether the model would classify a mostly indoor walk as indoor and vice versa. It is important to note that the GPS data can be noisy and interpretation can be difficult, even with the support of the self-report data (Fig. 3).

### Outdoor versus indoor walking analysis

Once we chose the best performing model, we classified the walking periods labeled indoor and outdoor and the unlabeled walking periods of dataset A, as well as all the walking periods in dataset B (Fig. 1). Then, we characterized the differences between indoor and outdoor walking periods. We looked at the differences in walking period duration and continuity, as well as an essential health indicator: walking speed.

#### Walking speed estimation

We used the method described in Baroudi et al.^41^ to estimate stride speed from the accelerometer. Briefly, this method leveraged the relationship between stride speed *v* and stride frequency *f*^42,43^:

2 $$\begin{aligned} v = \exp {\frac{\ln {(a\cdot f)}}{1-b}}, \end{aligned}$$

where *a* and *b* are model parameters. Stride frequency *f* can be accurately estimated from stride detection using the accelerometer and we identified in previous studies the parameters *a* and *b* for each subject in both datasets using a foot-worn inertial measurement unit^17,31^. Researchers reported that 97% of the stride speed error was under $0.2 \,{\text {m}} \cdot {\text {s}}^{-1}$ using this method. This framework can be used to estimate the relative speed differences for individuals walking in the real world using only an accelerometer.

#### Statistical analysis

Our goal was to understand the difference in walking speed, walking period duration, and continuity between indoor and outdoor settings, whilst taking into account the nested nature of our data: walking speeds are nested within walking periods, and walking periods are nested within participants. To tackle these inherent dependencies and repeated measures from each participant, we used linear mixed-effects models. This strategy facilitated the management of our multilevel data structure, properly adjusting for the correlations among multiple walking speeds, walking period duration, and continuity measures taken from the same walking period and participant. We built three models using walking speed, walking period duration, and walking period continuity as the dependent variables, while the indoor/outdoor condition was the fixed effect, and the walking period (for the walking speed model only) and participant identifiers were the random effects. We tested the assumption of normally distributed and homogeneous residuals by visualizing QQ-plots and residuals versus fitted values plots. We normalized walking speed by $\sqrt{g\cdot l_0}$, with $l_0$ being the participant’s leg length^34^. This design effectively captured the variability in walking period parameters both within and across periods and participants. The models were implemented in R using the ‘lme’ function from the ‘nlme’ package^44^. We explored different correlation structures for the random effects and selected the most appropriate model based on the Akaike Information Criterion (AIC)^45,46^. The model with the lowest AIC was deemed the best fit for our data. The estimated fixed effect for the condition serves as an indication of the expected change in parameters when transitioning from indoor to outdoor environments, while accounting for the nested structure of the data.

## Results

### Classification algorithm evaluation

#### Model validation

Tables 2 and 3 summarize the validation results for the different cases. The data from subjects 1, 4, 7, 17, and 19 were randomly selected to be kept for the test set. The training set contained between 957 and 1040 walking periods, with $67.9 \pm 4.4 \%$ (range) of indoor labels. The validation set contained between 29 and 112 walking periods, with $66.0 \pm 51.4 \%$ of indoor labels. This high range can be explained by the fact that participants had different habits and behavior. Most participants had mostly indoor walking periods, but for instance S6 had only 34.5% of indoor walks. The test set contained 339 walking periods and 78.8% of these periods were labeled indoor. The models perform better when using the biomechanics feature set. The average accuracy, F1-score, and AUROC increase of approximately 0.2 for both Ensemble SVM and Random Forest models from using all features to the biomechanics features only. Random Forest and Ensemble SVM perform comparably with both sets of features. Because Random Forest algorithms are easier to interpret and use, we chose the Random Forest algorithm with the biomechanics feature set to use with our test set. The model trained and validated using Random Forest and the biomechancs features with S8 held out performed the worst ($accuracy = 0.828$, $F1{\text {-}}score = 0.857$, $AUROC = 0.897$) while the one with S9 held out performed the best ($accuracy = 1.000$, $F1{\text {-}}score = 1.000$, $AUROC = 1.000$).

**Table 2:** Model validation with all features.

Held out subject	Accuracy		F1-score		AUROC	
	Ensemble SVM	Random forest	Ensemble SVM	Random forest	Ensemble SVM	Random forest
S2	**0.958**	0.938	**0.974**	0.960	0.909	**0.982**
S3	**0.911**	**0.911**	**0.949**	0.948	0.780	**0.975**
S5	0.789	**0.853**	0.853	**0.892**	0.807	**0.863**
S6	0.862	**0.879**	0.833	**0.851**	0.968	**0.982**
S8	**0.862**	**0.862**	**0.889**	**0.889**	**0.899**	0.887
S9	**1.000**	**1.000**	**1.000**	**1.000**	**1.000**	**1.000**
S10	0.897	**0.936**	0.927	**0.953**	0.963	**0.971**
S11	0.788	**0.859**	0.862	**0.913**	0.898	**0.943**
S12	**0.990**	**0.990**	**0.994**	**0.994**	0.971	**0.984**
S13	**0.863**	0.843	**0.851**	0.833	0.904	**0.957**
S14	**0.900**	0.867	**0.906**	0.875	**0.968**	0.955
S15	0.709	**0.722**	0.693	**0.711**	0.857	**0.921**
S16	0.907	**0.926**	0.944	**0.955**	0.825	**0.938**
S18	0.978	**0.989**	0.986	**0.993**	**0.979**	**0.979**
S20	**0.781**	**0.781**	**0.840**	**0.840**	**0.832**	0.830
Average	0.880	**0.890**	0.900	**0.907**	0.904	**0.945**

**Table 3:** Model validation with biomechanics features.

Held out subject	Accuracy		F1-score		AUROC	
	Ensemble SVM	Random forest	Ensemble SVM	Random forest	Ensemble SVM	Random forest
S2	0.917	**0.938**	0.947	**0.960**	0.894	**0.979**
S3	**0.893**	0.884	**0.939**	0.933	0.789	**0.878**
S5	**0.874**	0.863	**0.909**	0.901	0.848	**0.899**
S6	0.862	**0.879**	0.833	**0.851**	0.908	**0.986**
S8	**0.862**	0.828	**0.889**	0.857	0.846	**0.897**
S9	**1.000**	**1.000**	**1.000**	**1.000**	**1.000**	**1.000**
S10	0.885	**0.923**	0.919	**0.944**	0.905	**0.962**
S11	**0.941**	0.882	**0.966**	0.929	0.946	0.905
S12	**0.990**	0.962	**0.994**	0.976	**0.972**	0.968
S13	**0.863**	**0.863**	**0.851**	**0.851**	0.894	**0.909**
S14	0.950	**0.967**	0.958	**0.971**	0.958	**0.984**
S15	0.810	**0.848**	0.776	**0.806**	0.881	**0.934**
S16	**0.926**	0.889	**0.955**	0.930	0.909	**0.975**
S18	0.978	**0.989**	0.985	**0.993**	**0.999**	**0.999**
S20	**0.822**	**0.822**	**0.876**	0.874	0.838	**0.848**
Average	**0.905**	0.902	**0.920**	0.918	0.906	**0.941**

#### Model testing and choice

We evaluated the 15 models validated using Random forest and the biomechanics feature set on the 5-participant test set. We found that Model S16 (e.g., the model that was trained and validated with S16 held out) outperforms the other models, with an accuracy of 0.941, an F1-score of 0.963, and an AUROC of 0.931, which represented at least + 0.01 than the other models for all metrics (Table 4). As such, we chose this model to label the rest of the data and characterize the differences between indoor and outdoor walking periods.

**Table 4:** Model choice—Results of the 15 models with the 5-participant test set.

	Accuracy	F1-score	AUROC
Model S2	0.917	0.948	0.940
Model S3	0.917	0.948	0.935
Model S5	0.909	0.942	0.923
Model S6	0.920	0.950	0.921
Model S8	0.917	0.948	0.932
Model S9	0.929	0.956	0.918
Model S10	0.920	0.951	0.936
Model S11	0.917	0.948	0.924
Model S12	0.920	0.950	0.927
Model S13	0.926	0.954	0.926
Model S14	0.914	0.946	0.932
Model S15	0.932	0.958	0.917
**Model S16**	**0.941**	**0.963**	**0.931**
Model S18	0.923	0.952	0.930
Model S20	0.923	0.952	0.942
Best model	0.941	0.963	0.931

Model Sx corresponds to the models trained and validated with the leave-one-participant-out scheme with Sx held out using the Random Forest algorithm with the biomechanics features set as shown in Table 3. Model S16 performs the best, as it had the highest accuracy, F1-score, and AUROC.

#### Feature importance

**Figure 4:**
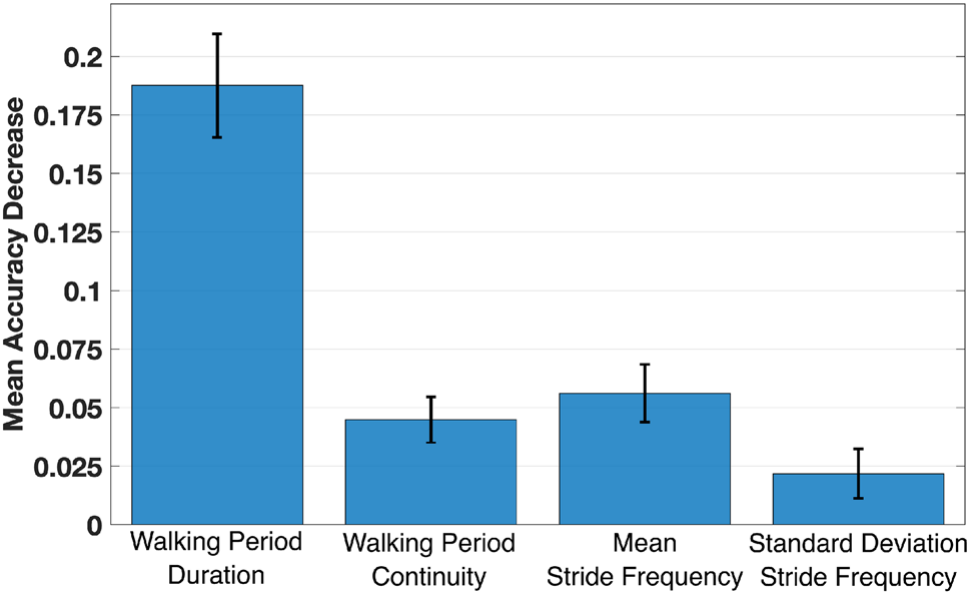
Feature importance using feature permutation—Decrease in model accuracy for each shuffled feature. The error bars represent standard deviation. A decrease in accuracy indicates that, when the given feature was perturbed (e.g., randomly shuffling its values), the performance of the model degraded. This means that this feature contains meaningful information on which the model relies to make its predictions.

We found that walking period duration was the most important feature in the model we chose (Fig. 4). When the values of this feature were shuffled within the validation set, it led to a decrease in model accuracy of 0.19. Walking period continuity, mean stride frequency, and standard deviation of stride frequency led to decreases in accuracy of 0.04, 0.06, and 0.02 respectively.

### Model classifications on mixed walking periods

**Figure 5:**
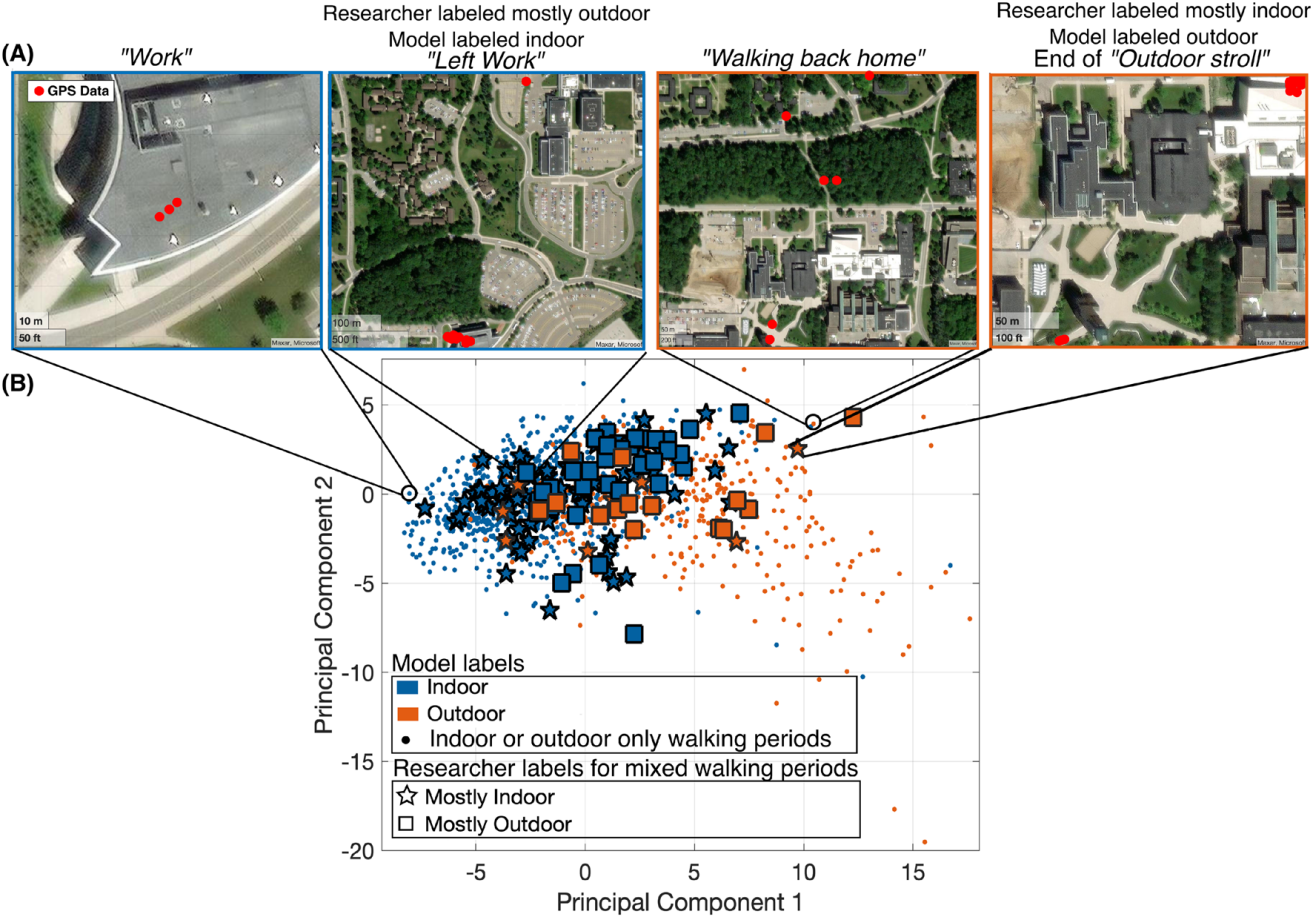
Analysis of mixed walks—(**A**) GPS and self-report data is shown for a sample of walks labeled by the model as indoor (left two images) and outdoor (right two images), as indicated by the coloring. The second and fourth images were originally labeled as mostly outdoor and mostly indoor by the researcher as indicated by the star and square shapes. The mixed walking periods highlighted here were mislabeled by the researcher. The sampling rate of the GPS data is variable and depends on the participant’s phone type. (**B**) Principal component analysis with the mixed walking periods colored based on the model classification. The shapes represent the researcher’s labels. We can see that the majority of mostly indoor walks are labeled indoor and vice versa, and that the walking periods that were mislabeled by the researcher are correctly labeled by the model.

Figure 5 shows a visualization of walking periods labeled mixed (e.g., indoor and outdoor) using principal component analysis with the walking periods labeled only indoor or outdoor. We observed that the majority of walks labeled mostly indoor clusters with the walks labeled only indoor and vice versa for outdoor walking periods. The model we chose classified 71% of mostly outdoor walks as outdoor and 88% of mostly indoor walks as indoor. Figure 5 also shows the self-report and GPS data from two walking periods. It is likely that the researcher incorrectly labeled these mixed walks, but the model managed to classify them more accurately as it reached good validation performances showing its learning of the characteristics of indoor versus outdoor walks.

### Characterization of indoor versus outdoor walking periods

After all walking periods were labeled, we had 69,616 stride speed values and 3766 walking periods from dataset A, compared to 53,930 stride speed values and 3701 walking periods from dataset B. The ratio of indoor versus outdoor was approximately 80:20 for both datasets. Different linear mixed models were built to evaluate the effect of context (e.g., walking environment) on walking speeds, walking period duration, and continuity. First, we found a large significant effect of context on walking speed $b = 0.095$, $t(117678) = 79.5$, $p < 0.001$. This indicates that normalized walking speed increases by 0.095 from indoor to outdoor walks (the normalized value of 0.095 corresponds to approximately $0.28 \,{\text {m}} \cdot {\text {s}}^{-1}$ depending on the participant’s leg length). We also found a large significant effect of context on walking period duration and continuity, $b = 9.25$, $t(7446) = 48.9$, $p < 0.001$ and $b = 20.14$, $t(7446) = 26.2$, $p < 0.001$. These results suggest that duration increases by 9.25 min and continuity by 20.14% from indoor to outdoor walks.

**Figure 6:**
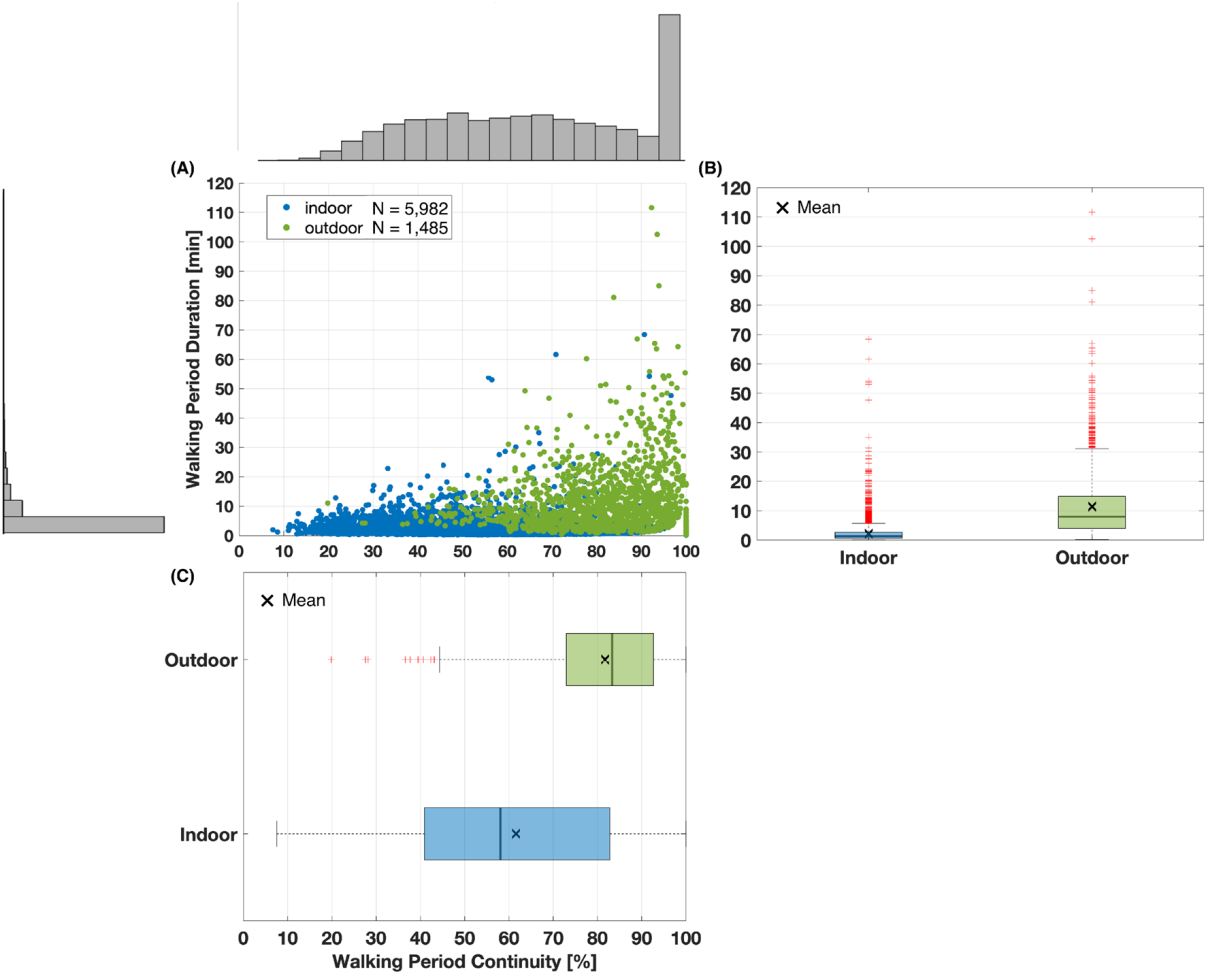
Indoor and outdoor walking periods duration and continuity—Relationship between walking period duration and continuity—(**A**) Scatter plot with marginal distributions of waking period duration and continuity. Each dot corresponds to a walking period. (**B,C**) We binned all walking periods by their context and looked at their durations and continuity.

**Figure 7:**
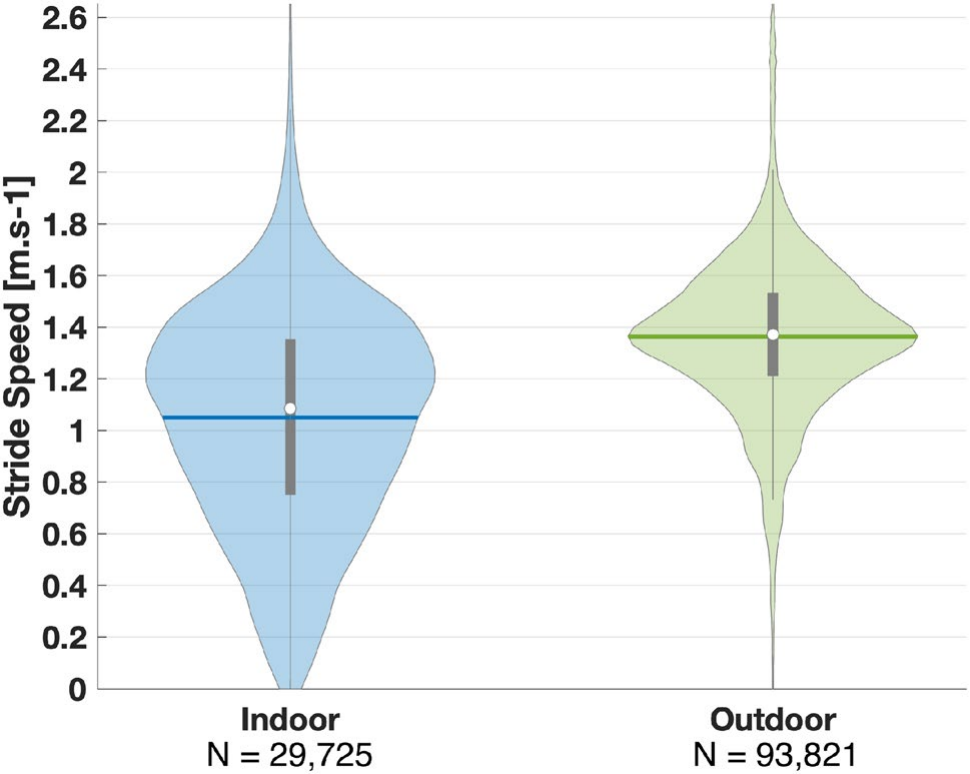
Stride speed for walking periods indoor vs. outdoor—Distribution of stride speeds for all participants grouped by context. Each shaded area represents the shape of the distribution, and the horizontal lines mark the mean.

These results are illustrated in Figs. 6 and 7. We observe that outdoor walking periods have overall higher duration ($\mu _{outdoor}$ = 11.4 min versus $\mu _{indoor}$ = 2.2 min) and continuity ($\mu _{outdoor} = 81.7\%$ versus $\mu _{indoor} = 61.6\%$). Participants walked on average $0.28 \,{\text {m}} \cdot {\text {s}}^{-1}$ faster when walking outdoor compared to indoor. We also observe a larger variability in the distribution of stride speed indoor compared to outdoor ($\sigma _{indoor} = 0.43 \,{\text {m}} \cdot {\text {s}}^{-1}$ versus $\sigma _{outdoor} = 0.31 \,{\text {m}} \cdot {\text {s}}^{-1}$).

## Discussion

In the real world, individuals exhibit great variability in walking patterns and are able to adapt to diverse environmental contexts. Understanding these contexts is essential for extracting meaningful information about a person’s mobility. Determining whether an individual is walking indoors or outdoors is a critical element of context, given the stark differences in environment and conditions that these two settings present. Here, we developed a novel framework that utilizes only an accelerometer to accurately classify indoor versus outdoor walks. To be able to ensure ecological validity from this reduced sensor set, we leveraged a unique dataset with an extended sensor suite that contained the accelerometer. Then, we used this approach to quantify the differences between indoor and outdoor walking patterns. This framework not only demonstrates the potential to use a minimal sensor suite to successfully gain important contextual information but also enables a more comprehensive understanding of real-world walking behavior.

Both Ensemble SVM and Random forest trained with different feature sets were able to learn the characteristics of indoor and outdoor walks. The performances across models were very high, as reflected by average accuracies, F1-scores, and AUROC exceeding 0.88, 0.90, and 0.86 respectively for all training scenarios (Tables 2, 3). Trained models performed better with the biomechanics feature set, suggesting that these features are sufficient to capture the inherent differences between indoor and outdoor walking periods (Table 3). Notably, walking period duration was the most important feature (Fig. 4) with outdoor walks being longer on average than indoor walks ($\sim + 9 \,{\text {min}}$) (Fig. 6). Average stride frequency was also an important feature, as outdoor walks tend to have a higher intensity than indoor walks. The high performance of the classifier also suggests that the grouping of walking bouts into walking periods is an effective representation of real-world walking^31^. We chose the best performing model that used the Random Forest algorithm trained with the biomechanics feature set (Table 4). Using the biomechanics feature set as opposed to the raw data enables the model to be reused with different sensor types and placements. In fact, stride frequency was chosen because it can easily be derived directly from various sensors, even from smartwatches^47–49^. The choice of Random Forest also increases the interpretability and generalizability of our model for other populations. As such, the model we developed could be extended to other studies of mobility and help improve the understanding of human data from wearable sensors.

We used the developed model to characterize the differences between walking indoor compared to outdoor using a large dataset. We found that outdoor walking periods were significantly longer, more continuous (e.g., less standing time), and had higher stride speed (Figs. 6, 7). Researchers have been increasingly interested in the measurement of walking speed in the real world, as it is a critical health indicator for various health issues^1,50–52^. Our observations show that individuals greatly vary their walking speed indoor (Fig. 7). On the other hand, individuals took more strides outdoor, with less variability in walking speed. This suggests that isolating outdoor walks could potentially improve estimates of preferred walking speed in the real world. This substantiates the findings that longer walks show greater discriminative power for clinical populations^53^. Understanding the proportion of activity spent indoor versus outdoor can also be useful for the improvement of physical activity. Although any increase in physical activity matters, there are proven benefits to walking outdoors^54–56^. As such, our models could be used in the different stages of intervention design, from the baseline physical activity assessment to the monitoring of intervention efficacy. The reduced sensor set also enables higher compliance (e.g., the degree to which users correctly and consistently wear the device as intended), which is essential for the reliability and validity of data collected in the real world.

While this study integrates fundamental elements of context for real-world walking, there are numerous other contextual factors that can have an impact on walking behavior and biomechanics. There are also nuances within indoor and outdoor walks, such as terrain type, that also induce changes in walking patterns^12,13^. Future work should investigate those factors and their relationships with motion to potentially integrate additional classes or sub-classes into the model we developed. Further, we developed our model with 20 young adults, who were mostly students. Populations with specific habits, like a nurse, would potentially show long walks indoors that could be mistaken for outdoor walks given the importance of walking period duration in our model. Additionally, individual habits may be affected by climate and thus geographical location. Thus, the accuracy and generalizability of our model can be improved by collecting a larger dataset with a more diverse population over an extended period. Additionally, the off-the-shelf system used for this work was designed to be placed on the thigh. The gait metrics and the activity classification algorithm were tuned to measurements made from this location. However, this particular placement can become inconvenient for users during extended measurement periods. Future research should explore the use of consumer-grade wearables like smartwatches. The feature set we derived for our classification algorithm can potentially be accurately obtained from sensors with different placements. Lastly, our method was mainly developed for offline classifications, in the scenario where data is retrieved and post-processed to gain information on an individual’s behavior. Expanding this framework for online classification should be pursued, for potential use in fields like rehabilitation or assistive robotics.

### Supplementary Information

Supplementary Table 5.

## Data Availability

The datasets used and analyzed during the current study are available from the corresponding author upon reasonable request.
