# Supplementary material for: Classification of human walking context using a single-point accelerometer
Source: Sci Rep. 2024 Feb 6;14:3039. doi: 10.1038/s41598-024-53143-8 (PMC10847110; doi:10.1038/s41598-024-53143-8)
Supplement: Supplementary file 1 — Supplementary Table 5. [file 41598_2024_53143_MOESM1_ESM.pdf]

Supplementary material

Table 5. List of Features - The first 4 features constituted the biomechanics feature set that was compared with the use of all features.

Signal used	Name	Formula	Meaning
Walking Period	Duration		Duration of walking period
	Continuity	$period\ continuity = \frac{period\ duration - standing\ time}{period\ duration} * 100$	Proportion of standing time in a walking period
Stride Frequency	Average	$\mu = \frac{1}{N} \sum_{t=1}^{N} \frac{1}{stridetime}$	Average of stride frequencies within a walking period
	Standard Deviation	$\sigma = \sqrt{\frac{1}{N-1} \sum_{t=1}^{N} \lvert \frac{1}{stridetime} - \mu \rvert^2}$	Standard deviation of stride frequencies within a walking period
	Kurtosis	$k = \frac{E(\frac{1}{stridetime} - \mu)^4}{\sigma^4}$	Measure of the weight of the distribution of stride frequencies within a walking period tails relative to its center
	Skewness	$s = \frac{E(\frac{1}{stridetime} - \mu)^3}{\sigma^3}$	Measure of the asymmetry of the distribution of stride frequencies within a walking period
	Min		Minimum value for stride frequencies within a walking period
	Max		Maximum value for stride frequencies within a walking period
3-axis Acceleration	Mean	$\mu = \frac{1}{N} \sum_{t=1}^{N} a_t$	Average of the signal
	Standard Deviation	$\sigma = \sqrt{\frac{1}{N-1} \sum_{t=1}^{N} \lvert a_t - \mu \rvert^2}$	Standard deviation of the signal
	Skewness	$s = \frac{E(a-\mu)^3}{\sigma^3}$	Measure of the asymmetry of the signal distribution
	Kurtosis	$k = \frac{E(a-\mu)^4}{\sigma^4}$	Measure of the weight of the signal distribution tails relative to its center
	Min		Minimum signal value
	Max		Maximum signal value
	Variance	$\frac{1}{N-1} \sum_{t=1}^{N} \lvert a_t - \mu \rvert^2$	Variance of the signal
	Mean Jerk	$\frac{1}{N} \sum_{t=1}^{N} \int a_t \, dt$	Average rate of change of signal with time, related to the signal smoothness
	Energy	$Energy = \sum_{t=1}^{N} a_t^2$	Measure of how "strong" the signal is over its duration
	Power	$Energy/N$	Total energy of the signal divided by its length
	Mean Absolute Deviation	$mad = \frac{1}{N} \sum_{t=1}^{N} \lvert a_t - \mu \rvert$	Measure of the dispersion of the signal
	Mean Frequency		Mean normalized frequency of the power spectrum of the time-domain signal
	Median Frequency		Median normalized frequency of the power spectrum of the time-domain signal
	Signal to noise ratio		Ratio between signal information and its background undesired noise
	Squared Root Mean Squared	$\mu = \frac{1}{N} \sum_{t=1}^{N} a_t^2$	Measure of the magnitude of the signal
	Integral of Power Spectral Density		Different measure of power, in the frequency domain, through the power spectral density
